# Mechanical factors play an important role in pectus excavatum with thoracic scoliosis

**DOI:** 10.1186/1749-8090-7-118

**Published:** 2012-11-12

**Authors:** Yuncang Wang, Gang Chen, Liang Xie, Jiming Tang, Xiaosong Ben, Dongkun Zhang, Pu Xiao, Haiyu Zhou, Zihao Zhou, Xiong Ye

**Affiliations:** 1 Department of Thoracic Surgery, The First Hospital of Hebei Medical University, Shijiazhuang, 050031, China; 2 Department of Thoracic Surgery, Guangdong Academy of Medical Sciences & Guangdong General Hospital, 106 Zhongshan Road, Guangzhou, 510080, China

**Keywords:** Pectus excavatum, Scoliosis, Mechanics, Image analysis

## Abstract

**Background:**

This study investigated the incidence, imaging characteristics and mechanical factors in scoliotic patients with pectus excavatum.

**Methods:**

A total of 142 scoliostic patients with pectus excavatum were evaluated prior to operation. The evaluation included a complete physical exam, phenotype and severity of the pectus excavatum, incidence and severity of scoliosis, and analysis of radiological images, including calculation of the Haller index.

**Results:**

Twenty five out of 142 patients (17.61%) with pectus excavatum had scoliosis with a Cobb angle >10 degrees, and in 80.00% of the cases the spinal column was bent to the right. Seventeen patients had bent-to-the-right spines that involved the 6th to 10 th thoracic vertebrae. We found that 23 out of 25 patients with a Cobb angle more than 10° were teenagers and adults. The incidence of scoliosis was only 6.06% in the children under 11 years whereas it was 21.79% in the teenage group.

**Conclusions:**

Mechanical forces appear to play a role in the coexistence of pectus excavatum and scoliosis. There is a relationship between age, severity (Haller index), asymmetry and scoliosis. The heart and mediastinum play a role in providing an outward force to the left of the sternum which may be an important reason for the coexistence of pectus excavatum and scoliosis, but the correlation needs further proof.

## Background

Pectus excavatum (PE), with a reported incidence of 0.1~0.3% [1], is a common chest wall deformity [2] caused by the excessive growth of the costal cartilage, producing a concave anterior chest wall [3]. The coexistence of pectus excavatum and scolosis has drawn much attention recently. Many reports have shown the coexistence of pectus excavatum and scoliosis in teenagers [4] and adults [5,6]. So far, the exact cause for the coexistence has remained unknown. In this retrospective study, we focused on the mechanical factors and aimed to investigate the role of the factors in the deformity.

## Methods

### Patients

A total of 142 scoliotic patients (120 males, 22 females) ranging in age from 3 to 32 years (mean, 14.0 ± 5.4 years) with pectus excavatum were enrolled in this study. All the patients underwent Nuss procedures in Guangdong Academy of Medical Sciences & Guangdong General Hospital between July 2007 and September 2010. We excluded all cases of Marfan Syndrome, Shprintzen Syndrome and Loeys-Dietz Syndrome. All patients gave their informed consent in writing and this study was approved by the ethics committee of Guangdong General Hospital.

### Measurement

All patients in the study had a preoperative computed tomography (CT) scan and a chest X-ray. The data related to thoracic scoliosis, Haller index and asymmetry of the right and lefty thoracic cavities Scoliosis direction were measured. For grouping purpose, we used letters A through L to categorize different groups (Tables 1, 2, 3).

**Table 1:** Thoracic scoliosis distribution of the patients with Cobb angle greater than 10°

	**Group A: Thoracic vertebra 1–5 (n=7)**	**Group B: Thoracic vertebra 6–10 (n=17)**	**Group C: Thoracic vertebra 11-lumbar vertebra 3 (n=1)**	***P***
Scoliosis bent to right (Percentage)	2 (28.57)	17 (100.00)*	1 (100.00)	<0.0005

*Compared with group A. Pearson χ2 analysis was used.

**Table 2:** Age distribution of the patients with Cobb angle greater than 10 °

	**Group D: ≤10Y (n=33)**	**Group E: 11~17Y (n=78)**	**Group F: ≥18Y(n=31)**	***P***
Scoliosis case (Percentage)	2 (6.06)	17 (21.79)*	6 (19.35)	<0.05

*Compared with group D.

**Table 3:** Haller index distribution of the patients with Cobb angle greater than 10°

	**Group G: <3.2 (n=19)**	**Group H: 3.2≤HI<3.5 (n=18)**	**Group I: ≥3.5 (n=105)**	***P***
Scoliosis case (Percentage)	2 (10.53)	3 (16.67)	20 (19.05)*	<0.05

*Compared with group H; HI: Haller Index.

The Haller index was created in 1987 by Drs. Haller, Kramer and Lietman [7]. The index is a mathematical relationship showing the ratio of the transverse diameter and the antero-posterior diameter:

(1) HallerIndex=distance1/distance2

Where distance 1 is the distance of the inside ribcage and distance 2 is the distance between the sternum and vertebrae. A normal Haller index is about 2.5. A surgery is warranted if the index is greater than 3.2. The mean Haller index reported by Kelly et al. was 5.15±2.32 [8].

The Cobb angle was originally used to measure coronal plane deformity on antero-posterior plain radiographs in classifying scoliosis [9]. In assessing sagittal plane deformity, the Cobb angle is defined as the angle formed between a parallel line to the superior endplate of one vertebra above the fracture and a parallel line to the inferior endplate of the vertebra one level below the fracture. As a general rule, a Cobb angle of 10 degrees is a minimum angulation for scoliosis.

### Date analysis

Data analysis was performed using Miscrosoft EXCEL, Bivariate analysis was done using Fisher’s Exact test (n<40) and Pearson χ2 analysis (n>=40) with an α value set at .05 for significance.

## Results

In 25 (17.61%) of the 142 pectus excavatum patients there was scoliosis with a Cobb angle greater than ten degrees. In this study, the prevalence of scoliosis in pectus excavatum patients was higher than that of elementary and high school students in Guangzhou and Xi'an. In this study, spines of 20 (80.00%) of the 25 patients bent to the right. We found that in 2 patients whose spines bent to the right the scoliosis involved the 1st through 5th thoracic vertebrae (group A). We found that in 17 patients whose spines bent to the right, the scoliosis involved the 6th through 10th thoracic vertebrae (group B). There was a significant difference between group A and group B (p<0.0005, Table 1).

We found that 23 out of 25 patients with a Cobb angle greater than 10° were teenagers and adults. The incidence of scoliosis was only 6.06% in the children under 11 years (group D) whereas it was 21.79% in the teenage group (group E), and 19.35% in the adult group (group F). There was a significant difference between group D and group E (P < 0.0005, Table 2).

With regard to the severity of the pectus excavatum all the cases associated scoliosis (Cobb angle> 10°) were in the group with a Haller index greater than 3.2 but less than 3.5 (16.67%) (group H). In the other group with a Haller index equal or greater than 3.5 (group I) , the incidence of scoliosis was 19.05%. A significant difference was found between group H and group I (P < 0.05, Table 3). And we knew that the incidence of scoliosis was only 10.53% in the group with a Haller index less than 3.2 (group G) (Table 3).

## Discussion

In this study, the incidence of scoliosis with a Cobb angle greater than 10° was 17.61% in our pectus excavatum patients and it was much higher than that in ordinary population. One characteristic feature we found was that in 80.00% of the cases the spinal column bent to the right, involving the thoracic through the 3rd lumbar vertebrae. We also found that if the scoliosis included the 6th to 10th thoracic vertebrae, 100% of the patients (17 cases) had their spine bent to the right. We found a correlation among scoliosis, age and severity, which was measured by Haller index.

The incidence of thoracic scoliosis was associated with pectus excavatum. Although we excluded patients having Marfan’s disease and other connective tissue disorders, the incidence of scoliosis with a Cobb angle> 10° was much higher than that in ordinary population, yielding 17.6% in our pectus excavatum patients. Akcali et al. [5] reported that scoliosis was associated with pectus deformities in 13.5% of his surveyed students recruited from public schools in Manaus, Brazil [10]. The scoliosis incidences discovered in teenagers during routine check-ups in Guangzhou and Xi’an were 1.07% and 1.33%, respectively [11,12]. Then the question was why the incidences between different populations were different? Genetic or familial factors might contribute to the disease [13-15]. Biomechanical factor might be involved as well. We noticed that in 80.00% of the cases the spine bent to the right and this phenomenon was especially evident if the scoliosis involved the 6th to 10th thoracic vertebrae, where the heart is located. We speculated that pectus excavatum pushed the heart to the left and counterforce generated by the heart pushed the thoracic vertebrae to the right. The anterior chest wall might push the heart to the left, rotating the mediastinum as the heart moves over. The chest continues to retract on the right side causing the mediatinum and vertebrae to be pulled over to the right resulting in an asymmetric pectus excavatum and scoliosis to the right. The beating heart also contributes to the forces causing the vertebrae to bend to the right. After closely examining the difference between Group H and Group I, we found that adolescent patients had higher incidence than teenage patients (see Table 3), suggesting the deformity progresses with age. Scoliosis with a Cobb angle >10 was rare in the children under 11 years but became much more common in teenagers and adults (21.79%, 19.35%). The cases of Cobb angle greater than 10 ° were mainly distributed in the severe group with a Haller index equal or greater than 3.5 (19.05%).

## Conclusion

In conclusion, mechanical factor plays an important role in the coexistence of pectus excavatum and thoracic scoliosis. We have found a correlation among scoliosis, age and severity. However, the underlying mechanism needs to be further investigated. Future treatment should take the mechanical factor into consideration.

## Competing interests

The authors declare that they have no competing interests.

## Authors’ contributions

YW participated in the surgeries and wrote the manuscript. GC led and performed the surgeries. Other authors participated the surgeries and collected the data. All authors read and approved the final manuscript.
